# A New Fishfly Species (Megaloptera: Corydalidae: *Neohermes* Banks) Discovered from North America by a Systematic Revision, with Phylogenetic and Biogeographic Implications

**DOI:** 10.1371/journal.pone.0148319

**Published:** 2016-02-17

**Authors:** Xingyue Liu, Shaun L. Winterton

**Affiliations:** 1 Department of Entomology, China Agricultural University, Beijing, 100193, China; 2 California State Collection of Arthropods, California Department of Food & Agriculture, Sacramento, California, United States of America; University of Arkansas, UNITED STATES

## Abstract

The taxonomy of Megaloptera from the Nearctic region is fairly well known and their faunal diversity has been largely surveyed, even in relatively remote regions. However, the evolutionary history of Nearctic Megaloptera is still poorly known with phylogenetic and biogeographic studies lacking. In this paper, we report a new fishfly species of the endemic North American genus *Neohermes* Banks, 1908, increasing the total number known of species to six. This new species (*Neohermes inexpectatus* sp. nov.) is currently known to occur only in California (USA) and is apparently confined to the Northern Coastal Range. The new species resembles the three *Neohermes* species from eastern North America based on the relatively small body size and the presence of female gonostyli 9. However, our phylogenetic analysis using adult morphological data recovered the new species as the sister species to the remaining *Neohermes*, which includes two species from western North America and three from eastern North America. According to the present interspecific phylogeny of *Neohermes*, with reconstructed ancestral areas, the initial divergence within the genus was found to take place in western North America, with a subsequent eastward dispersal. This likely lead to the modern distribution of *Neohermes* in eastern North America with the closure of the Mid-Continental Seaway, which separated western and eastern North America in the Mid-Late Cretaceous (100–80 MYA) and finally disappeared at the end of the Cretaceous (70 MYA). The uplift of the Cordilleran System probably accounted for the divergence between the eastern and two western *Neohermes* species.

## Introduction

The insect order Megaloptera is one of the primitive holometabolous groups with the origin dating back at least in the late Permian [1]. Modern Megaloptera include dobsonflies (Corydalidae: Corydalinae), fishflies (Corydalidae: Chauliodinae) and alderflies (Sialidae), comprising more than 380 species represented unevenly in all major biogeographical regions [2,3]. Despite the relatively small number of species, Megaloptera (particularly Corydalidae) are well known insects readily found in general entomological collections because of their large body size and frequent bizarre external appearance, e.g., conspicuously large mandibles in some males. The larvae of Megaloptera are aquatic and inhabit various freshwater habitats (usually clean streams, rivers, ponds, etc.) where they are predaceous on other benthic macroinvertebrates. They are valuable components in aquatic ecosystems especially for fisheries and angling in North America, or consumed as local food and medicine in some Asian countries, as well as widely used in freshwater biomonitoring for stream health [3,4]. Megaloptera are of particular interest for phylogenetic and biogeographic studies due to their apparent primitive morphology and disjunct geographic distributions. Hence, the taxonomy of Megaloptera has been well studied and most of the world species have been described or re-described in a modern approach by virtue of several neuropterologists, e.g. Ross (American Sialidae) [5], Flint (American Chauliodinae) [6,7], Aspöck et al. (European Sialidae) [8], Vshivkova (Caucasus and Siberian Sialidae) [9], Theischinger (Australian Megaloptera) [10], Contreras-Ramos (Neotropical Corydalinae and Sialidae) [11–13], Yang & Liu (Chinese Megaloptera) [2], Liu et al. (southeastern Asian Megaloptera) [14–16], and Liu et al. (African Megaloptera) [17–19].

The North American Megaloptera comprise 45 species (1 genus and 3 species of Corydalinae, 6 genera and 18 species of Chauliodinae, and 2 genera and 24 species of Sialidae), representing approximately 1/10 of the world megalopteran fauna [2,20]. It is noteworthy that there is a high percentage of endemic fishfly genera and about one third of world alderfly species in North America. The diversity of North American Megaloptera is well known based on long-term taxonomic studies and field investigations. The most recently discovered species in North America are *Protochauliodes cascadius* Evans (Corydalidae), described in 1984 [21], and *Sialis bilobata* Whiting (Sialidae), described in 1991 [22].

In this paper, we report another new species (*Neohermes inexpectatus* sp. nov.) of Corydalidae, discovered in California (USA). The genus *Neohermes* is one of the most distinctive genera of Chauliodinae, distinguished by the sexually dimorphic antennae (moniliform with whorl of long setae and more than 3/4 of forewing length in male, but filiform with much shorter setae and less than 1/2 of forewing length in female). The adult is also characterized by the forewing 2A with anterior branch fused with the stem of 1A for a short distance and the distally forked posterior branch of Rs. Five species of *Neohermes* have been described previously: *Neohermes californicus* (Walker, 1853), *Neohermes filicornis* (Banks, 1903), *Neohermes concolor* (Davis, 1903), *Neohermes angusticollis* (Hagen, 1861), and *Neohermes matheri* (Flint, 1965); four of which are endemic to United States. Interestingly, *Neohermes* has a disjunct distribution in North America, with distinct and widely separated groups of western and eastern species. *Neohermes californicus* and *N*. *filicornis* occur in western United States and northwestern Mexico, while the remaining three species are restricted to eastern United States. *Neohermes californicus* and *N*. *filicornis* can be easily distinguished from the three eastern counterparts by the much larger body size and denser forewing markings. However, the male genitalia provide more important diagnostic characters to distinguish species of *Neohermes*, although intraspecific variation of male genitalia, particularly the male ectoprocts, is present in *N*. *concolor* and possibly also in some other species [23].

We present a phylogeny of *Neohermes* based on adult morphological characters to reveal the systematic position of the new *Neohermes* species in the genus. Furthermore, we reconstructed the ancestral distribution areas in the phylogeny of *Neohermes*. The result sheds new light on the biogeography of Megaloptera.

## Results

### Taxonomy

#### *Neohermes* Banks

*Neohermes* Banks, 1908: 29 [24]. Type species: *Chauliodes filicornis* Banks, 1903: 238 [25], original designation.

**Fig 1:**
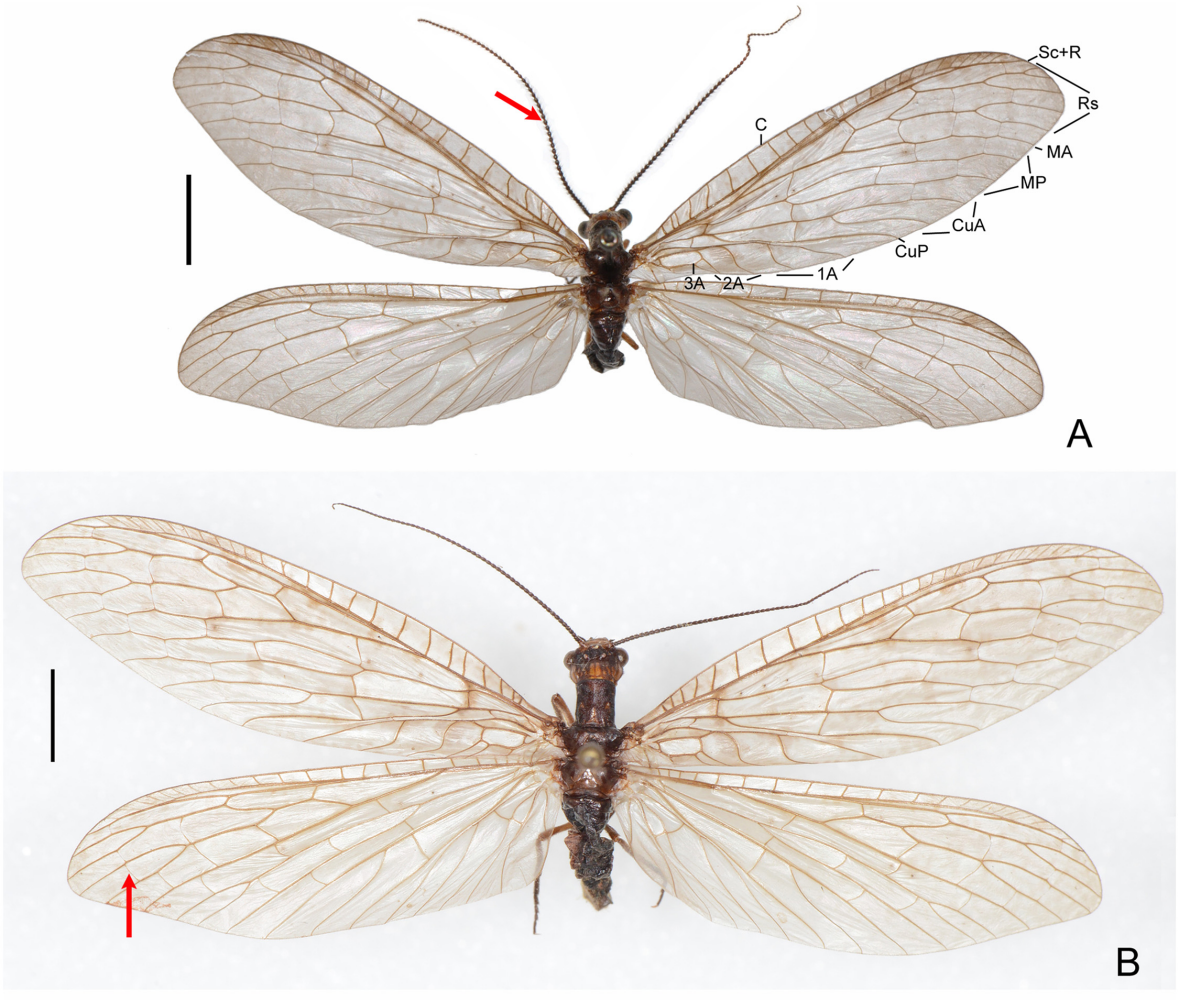
Habitus images of *Neohermes inexpectatus* sp. nov. A, holotype, male; B, paratype, female. Arrows indicate moniliform male antennae and presence of a crossvein beyond fork of posterior Rs branch. Scale bar = 5.0 mm.

**Fig 2:**
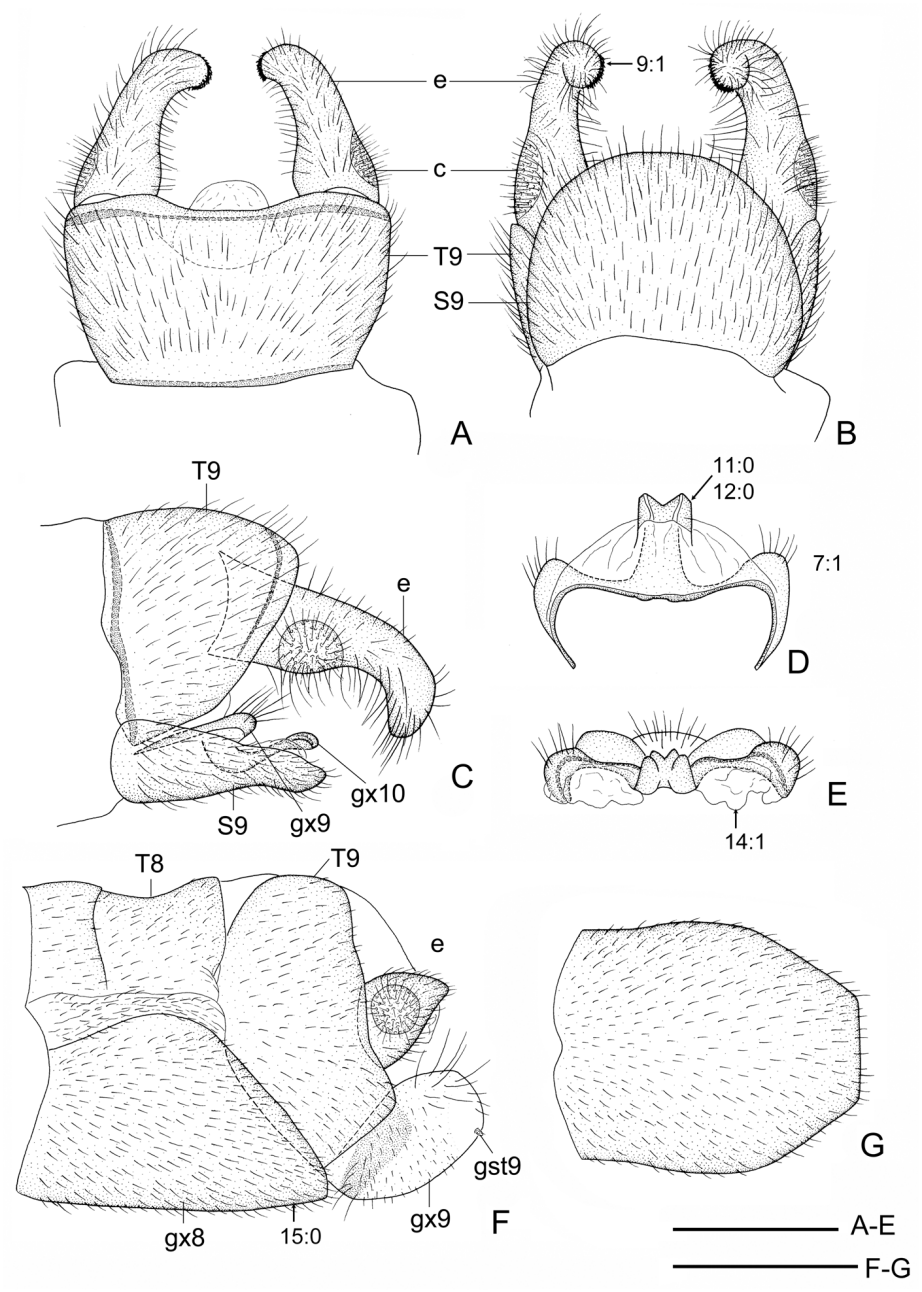
*Neohermes inexpectatus* sp. nov. A, male genitalia, dorsal view; B, male genitalia, ventral view; C, male genitalia, lateral view; D, male fused gonocoxites 10, ventral view; E, male fused gonocoxites 10, caudal view; F, female genitalia, lateral view; G, female fused gonocoxites 8, ventral view. S: sternite, T: tergite, gx: gonocoxite, gst: gonostylus, c: callus cercus, e: ectoproct. Arrow indicates character state used in the present phylogenetic analysis. Scale bar = 1.0 mm.

**Fig 3:**
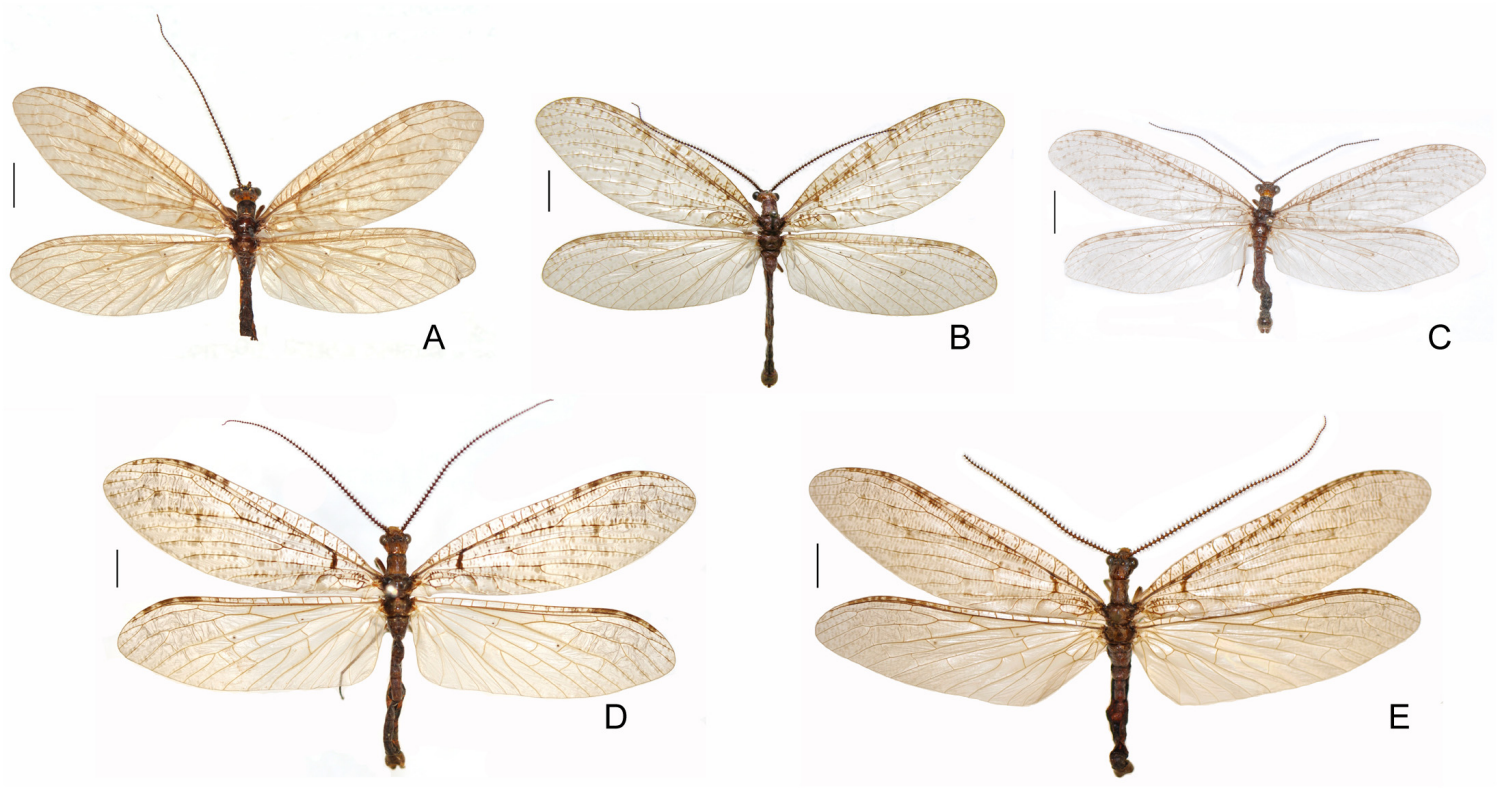
Habitus images of *Neohermes* spp. A, *N*. *angusticollis*, male; B, *N*. *concolor*, male; C, *N*. *matheri*, male; D, *N*. *californicus*, male; E, *N*. *filicornis*, male. Scale bar = 5.0 mm.

**Fig 4:**
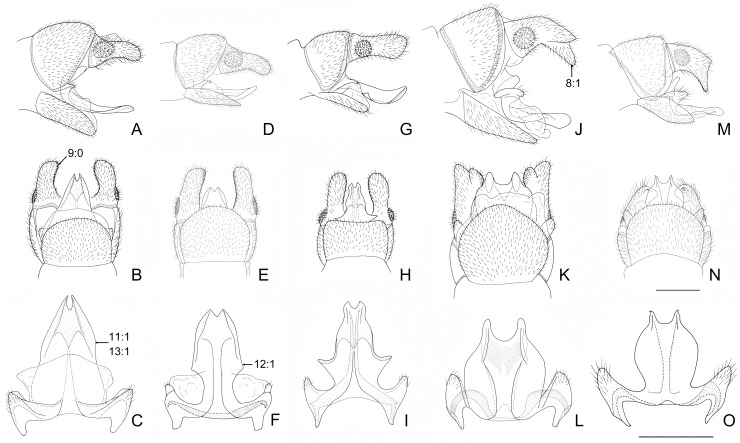
Male genitalia of *Neohermes* spp. A-C, *N*. *angusticollis*; D-F, *N*. *concolor*; G-I, *N*. *matheri*; J-L, *N*. *californicus*; M-O, *N*. *filicornis*. Upper: genitalia, lateral view; Middle: genitalia, ventral view; Lower: fused gonocoxites 10 with reduced gonocoxites 9, ventral view. Arrow indicates character state used in the present phylogenetic analysis. Scale bar = 1.0 mm.

**Fig 5:**
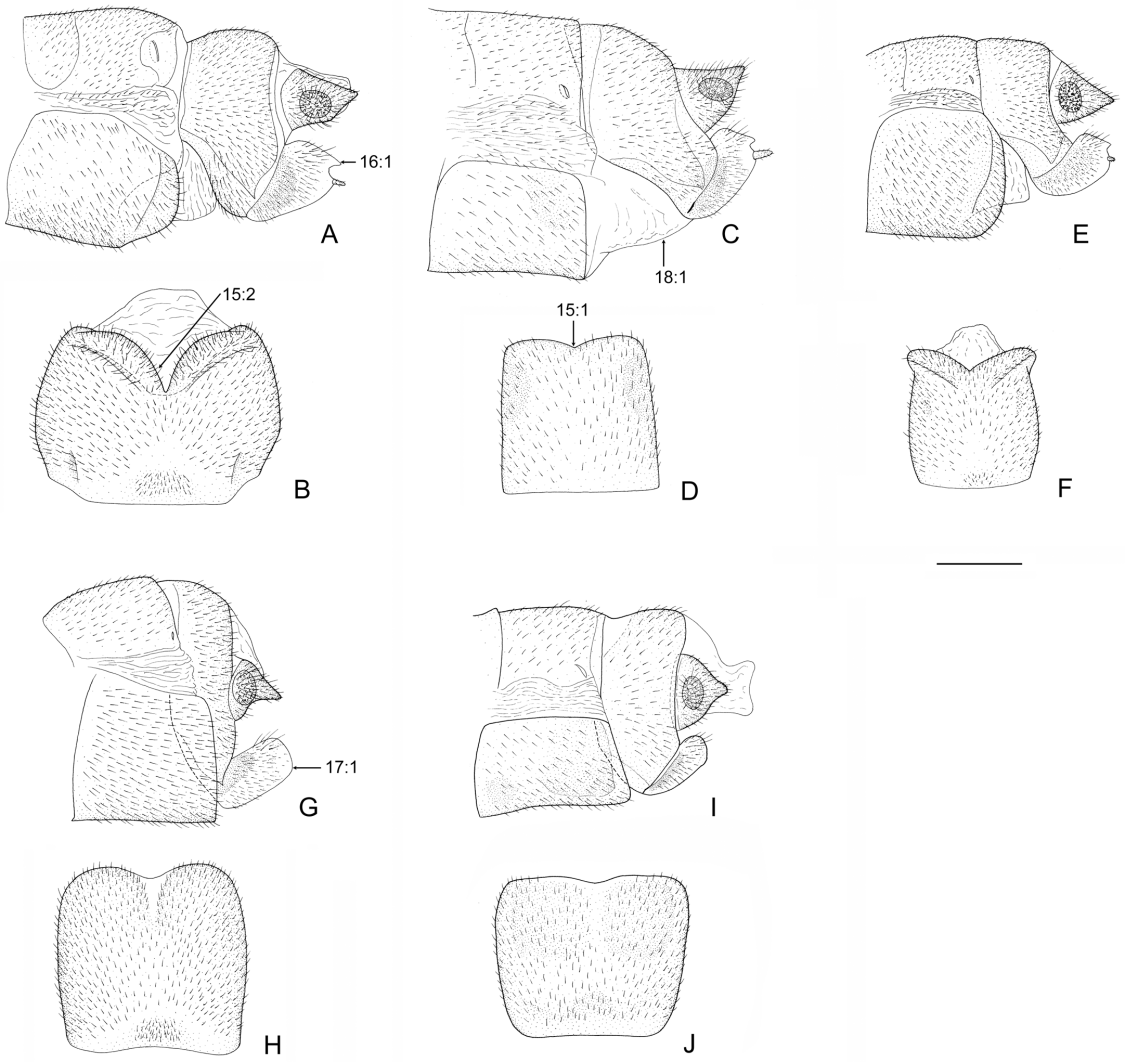
Female genitalia of *Neohermes* spp. A-B, *N*. *angusticollis*; C-D, *N*. *concolor*; E-F, *N*. *matheri*; G-H, *N*. *californicus*; I-J, *N*. *filicornis*. Lateral view of genitalia and ventral view of fused gonocoxites 8 are shown. Arrow indicates character state used in the present phylogenetic analysis. Scale bar = 1.0 mm.

Diagnosis. Adult (Figs 1–5). Medium to large-sized (male forewing length 26–50 mm). Body generally grayish, reddish or blackish brown. Antennae sexually dimorphic; male antenna moniliform with whorl of long setae, ca. 3/4 length of forewing, female antenna filiform with much shorter setae than male, ca. 1/2 length of forewing. Distance between lateral ocelli ~3.0 times as long as width of median ocellus. Wings narrowly elongated, with a few or numerous dark spots along longitudinal veins on forewing. Both anterior and posterior branches of Rs forked distad, a crossvein usually present between secondary distal branches of posterior Rs branch; three crossveins present between R and Rs; MA simple; MP with two simple branches; 1A 2-branched, with posterior branch sinuate; 2A 2-branched, with anterior branch sinuate and fused with stem of 1A for a short distance on forewing, and with posterior branch short and straight; base of MA on hindwing long, oblique, simply connecting to Rs. Male genitalia: Tergite 9 subquadrate, thick apodeme present along anterior and posterior margins; sternite 9 broad with arcuately convex posterior margin; gonocoxite 9 present, fused with lateral arms of fused gonocoxites 10; ectoproct stout, sometimes strongly curved ventrad or with additional ventral lobes, inner portion with numerous black rhabdoid-shaped setae; cercus rounded and feebly protruded; gonocoxite 10 strongly sclerotized, lateral arm subtriangular, median plate usually ovoid or blade-shaped with a notch at tip; a pair of membranous sac-like lobes protruding beneath anteroventral portion of fused gonocoxites 10; a setose patch present beneath anus. Female genitalia: gonocoxite 8 broad; gonocoxite 9 nearly rectangular, with or without gonostylus 9; ectoproct subtriangular, with round and feebly prominent cercus.

Larva. Head yellow with dark markings or blackish brown with a few yellow markings. Prothorax dorsally yellow with dark markings; meso- and metathorax dark brown with a few yellow markings. Antenna with distal two segments combined as long as second segment. Labrum nearly rectangular, ca. 2.0 times as wide as long. Terminal pair of abdominal spiracles opening at tips of rather short and coniform projections. See more details in Evans [26].

Biology. The biology of *Neohermes* is relatively better studied than many other fishfly genera, especially with detailed information on the life history of *N*. *californicus* [26]. The larvae of *N*. *californicus* and *N*. *filicornis* usually inhabit rocky intermittent or permanent warm streams, while the larvae of *N*. *concolor* were reported to live under leaf litter near a spring seep [26,27]. The life cycle for *N*. *californicus* was estimated to take two years in permanent warm water streams, but it is extended in intermittent streams where the water flows only during the winter months [26]. In drought streams, the larvae burrow into the stream bed and form a small chamber for diapause which may last for more than six months [26].

Remarks. Due to similar wing marking patterns and wing venations, *Neohermes* is closely related to *Protochauliodes*, and the females of these two genera are frequently misidentified with each other due to lack of distinct morphological differences [26]. The male antennae are different between these two genera and could be the most readily diagnostic morphological feature. When the female antennae are examined closely, the flagellomere is ovoid, more pilose in *Neohermes* but subtrapezoid and less pilose in *Protochauliodes*. Evans [26] noted that the presence/absence of a crossvein beyond the fork of posterior branch of Rs is another good character to distinguish *Neohermes* and *Protochauliodes* in most cases. However, it should be noted that this crossvein is absent only in the North and South American species of *Protochauliodes*, but it is present in the Australian *Protochauliodes* species. In addition, the female gonostylus 9 is absent or slender digitiform in *Neohermes*, but it is always robust in *Protochauliodes*.

#### *Neohermes inexpectatus* sp. nov.

urn:lsid:zoobank.org:act:51113617-733C-4D58-AD2F-84D34F45F45F

(Figs 1 and 2)

Description. Male. Body length 19 mm; forewing length 26 mm, hindwing length 23 mm.

Head dark brown, with yellowish brown clypeus; vertex with yellowish brown scars. Compound eye blackish brown; ocelli pale yellow, medially margined black. Antenna blackish brown, with distal half of flagellum slightly paler. Mouthparts brown; mandibles with distal half reddish brown, distal three segments of maxillary and labial palpi much darker.

Thorax blackish brown; pronotum medially with a pair of yellowish brown spots. Legs pale brown, with tibiae and tarsi black, bearing dense brownish setae; tarsal claws pale reddish brown. Wings pale smoky brown, pterostigmatic area pale brown. Forewing with indistinct, scattered brownish markings, which are slightly enlarged and darker on basal mp-cu crossvein and anterior branch of 2A. Hindwing immaculate. Veins brown.

Abdomen blackish brown. Tergite 9 (Fig 2A) subquadrate in dorsal view, slightly widened posteriad, with truncate anterior margin and slightly concaved posterior margin. Sternite 9 (Fig 2B) slightly longer than tergite 9, nearly semicircular in ventral view, with arcuately convexed posterior margin. Ectoproct (Fig 2A–2C) nearly as long as tergite 9, digitiform, distally curved inward and ventrad, with short spinous setae confined at tip. Gonocoxite 10 (Fig 2D and 2E) short, entirely concealed beneath sternite 9; median plate in ventral view narrowly rectangular, posteriorly with a V-shaped incision, and ventrally with a pair of longitudinal ridges; lateral arm long, incurved, anteriorly ending acutely, posteriorly roundly prominent and setose.

Female. Body length 20–27 mm; forewing length 29–33 mm, hindwing length 25–29 mm.

Fused gonocoxites 8 (Fig 2F and 2G) subtrapezoidal in lateral view, posterior margin strongly convexed, medially truncate in ventral view. Gonocoxite 9 (Fig 2F) broadly ovoid, posteroventrally with a tiny gonostylus 9. Ectoproct (Fig 2F) subtriangular, with slightly arched dorsal margin in lateral view.

Type material. Holotype ♂, “Cal[ifornia]., Sonoma Co[unty]., Camp Meeker [ca. 38°25'N, 122°57'W], VII.4.1950/H.H. Keifer Collector/*Protochauliodes minimus* (Davis, 1903) det. N. Penny, 1999” (CSCA). Paratypes 1♀, “U.S.A., CAL[IFORNIA]., Napa Co[unty]., N[orthern]. side Howell Mt., 2 mi. NNE. Angwin. [ca. 38°34'N, 122°26'W], 1300 ft [= 396 m], 8.VI.1978, H.B. Leech/At light/CASENT 8145994” (CASC); 1♀, “Calif[ornia]., Napa Co[unty]., Angwin, Oct. 20, 1975/Collected by L. Eighme/Pacific Union Colleage Collection donated to California Academy of Sciences—1989 thru Lloyd E. Eighme/CASENT 8149515” (CASC).

Distribution. U.S.A. (California).

Etymology. The specific epithet refers to the unexpected discovery of this new species.

Remarks. The holotype of *N*. *inexpectatus* sp. nov. bears an identification label “*Protochauliodes minimus* (Davis, 1903) det. N. Penny, 1999” given by Norman D. Penny (California Academy of Sciences) who is an eminent expert of New World Megaloptera and Neuroptera. However, the male moniliform antennae with whorl of long setae in this new species suggest its definite affiliation in *Neohermes*. Possibly, the misidentification was due to the similarly small body size and external appearance of the new species with various Californian *Protochauliodes* species in the same collection.

Compared with the other western North American fishfly species that are present in the collections with relatively large number of specimens, only one male and two females are known for the new *Neohermes* species. All the specimens of *N*. *inexpectatus* sp. nov. were collected from 1950s to 1970s and no specimen has been collected since then. Based on the collecting data, *N*. *inexpectatus* sp. nov. might be restricted to the Northern Coastal Range, and the flight period lasts at least from June to October.

### Key to species of *Neohermes*

1. Forewing length 29–50 mm in males and 36–54 mm in females; forewing with dense dark spots along longitudinal veins, arranging fine striated pattern (Fig 3D and 3E); male ectoproct distally with a ventral projection (Fig 4J and 4M); female gonostylus 9 absent (Fig 5G and 5I)…………………………………………………………………………………………………….2
  - Forewing length 26–32 mm in males and 29–39 mm in females; forewing with dark spots along longitudinal veins distantly apart or largely reduced (Figs 1 and 3A–3C); male ectoproct without ventral projection (Fig 4A, 4D and 4G); female gonostylus 9 present (Figs 2F, 5A, 5C and 5E)………………..……3
2. Male ectoproct with a ventral projection at tip (Fig 4M); female ectoproct feebly produced posteriad (Fig 5I); **western U.S.A. (AZ, CA, NM) and northwestern Mexico**……..*N*. *filicornis*
  - Male ectoproct with a ventral projection subdistad, forming a bifurcated apex (Fig 4J); female ectoproct strongly produced posteriad (Fig 5G); **western U.S.A. (CA, NV, OR)**….*N*. *californicus*
3. Forewing with indistinct dark markings (Fig 1); male ectoproct with apex distinctly curved ventromesad (Fig 2A); male fused gonocoxites 10 with median plate short, rectangular, entirely concealed beneath sternite 9 in ventral view (Fig 2D); female fused gonocoxites 8 subtrapezoidal in lateral view, with posterior margin strongly convexed (Fig 2G–2E); female gonocoxite 9 posteriorly without subtriangular projection (Fig 2G); **western U.S.A. (CA)**………………………………………………………………….…….*N*. *inexpectatus* sp. nov.
  - Forewing with distinct dark markings; male ectoproct with apex not or only feebly curved ventromesad (Fig 4B); male fused gonocoxites 10 with median plate much longer, blade-shaped, posteriorly extending beyond posterior margin of sternite 9 (Fig 4B); female fused gonocoxites 8 subquadrate in lateral view, with posterior margin concaved (Fig 5B); female gonocoxite 9 posteriorly with a small subtriangular projection (Fig 5A); **eastern U.S.A.**…………………..….4
4. Median plate of male fused gonocoxites 10 strongly prolonged and subtriangular (Fig 4C); female fused gonocoxites 8 distinctly concaved on posterior margin (Fig 5B)………………..….5
  - Median plate of male fused gonocoxites 10 feebly prolonged and sagittiform (Fig 4F); female fused gonocoxites 8 feebly concaved on posterior margin (Fig 5D); **widespread in eastern U.S.A.**…………………………………………………………………………………..*N*. *concolor*
5. Wings slightly grayish; median plate of male fused gonocoxites 10 anterolaterally with a pair of sharply pointed projections, posteriorly with a broad incision, leaving a pair of short and broad projections (Fig 4I); **southeastern U.S.A. (MS)**………………………………………..*N*. *matheri*
  - Wings slightly darkened, pale brown; median plate of male fused gonocoxites 10 anterolaterally feebly prominent, posteriorly with a deep, narrow incision, leaving a pair of narrow and sharply tapered projections (Fig 4C); **southeastern U.S.A. (GA, SC and NC)**………….*N*. *angusticollis*

### Phylogenetic analysis

**Fig 6:**
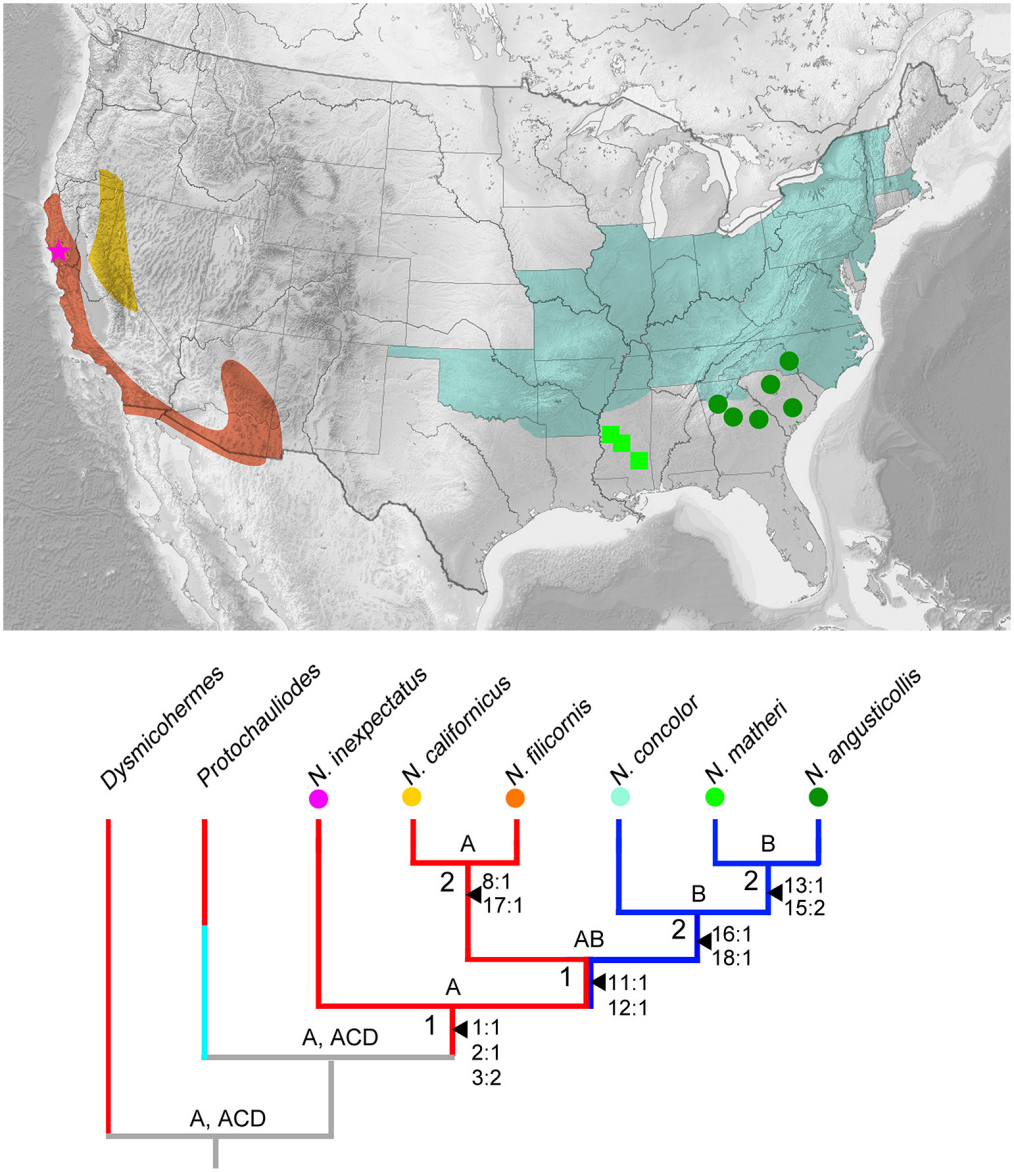
Interspecific relationships and reconstruction of ancestral areas for *Neohermes* species. Topology shown based on the most parsimonious tree from analyses with NONA and TNT. Bremer support values are shown at nodes. Only unambiguous, unique character changes are shown aside relevant branches. The areas are scored as follows: (A) western North America; (B) eastern North America; (C) southern South America; (D) eastern Australia. Colored branches indicate occurrence of relevant taxa in scored areas, i.e., A (red), B (dark blue), and C and D (bright blue). Colored circles aside *Neohermes* species link the distribution areas in the map (Reprinted from Relief location map of the USA (without Hawaii and Alaska) [28] under a CC BY license, with permission from Wikimedia Commons, the free media repository, original copyright 2010) upper to the phylogenetic tree. Distribution areas mainly follow the records from Flint [6], Evans [26] and Bowles and Mathis [23]. Purple star: *N*. *inexpectatus* sp. nov.; dark green circles: *N*. *angusticollis*; pale blue area: *N*. *concolor*; pale green squares: *N*. *matheri*; yellow area: *N*. *californicus*; orange area: *N*. *filicornis*.

The heuristic search in NONA yielded single most parsimonious tree (MPT) (length = 22, consistency index = 0.90, retention index = 0.88) (Fig 6), and the analysis in TNT also yielded the same phylogenetic tree (length = 22). The three antennal features support the monophyly of *Neohermes*. Within *Neohermes*, *N*. *inexpectatus* sp. nov. was assigned to be the sister to the remaining five species that form a monophyletic group supported by the prolonged median plate of male gonocoxites 10 with convex lateral margins (chars. 11:1 and 12:1). In this group, the species from western and eastern North America respectively form a monophyletic clade. The synapomorphies supporting *N*. *californicus* + *N*. *filicornis* are the male ectoproct distally with a ventral projection (char. 8:1) and the loss of female gonostylus 9 (char. 17:1). Whereas, two female characters, i.e. the gonocoxite 9 posteriorly with a small sub-triangular projection (char. 16:1) and the presence of broad membrane between gonocoxites 8 and abdominal segment 9 (char. 18:1), were assigned as the synapomorphies for the clade comprising the three eastern species. *N*. *angusticollis* and *N*. *matheri* are sister species with two synapomorphic characters: the prolonged sub-triangular median plate of the male gonocoxites 10 (char. 13:1) and the strongly concave posterior margin of female gonocoxites 8 (char. 15:1).

### Ancestral area reconstruction

Western North America was reconstructed to be the ancestral area for the initial divergence of *Neohermes* (Fig 6). Whole North America was assigned to be the ancestral area for the split between two western species (*N*. *californicus* and *N*. *filicornis*) and remaining three eastern species. Again, western North America was assigned to be the ancestral area for the divergence between *N*. *californicus* and *N*. *filicornis*, while eastern North America was the ancestral area for the sequential divergence of the three eastern species.

## Discussion

*Neohermes inexpectatus* sp. nov. represents the only new species of Megaloptera discovered in North America in the past 20 years. It is a remarkable supplement to the fauna of the North American endemic fishfly genus *Neohermes* since the last species of this genus was described 50 years ago. Surprisingly, the known distribution area of *N*. *inexpectatus* sp. nov. is just *ca*. 60 kilometers away from San Francisco; all examined specimens were collected in 1950-1970s and its current status and habitat condition is unknown. More importantly, the new species, as assigned in the present phylogenetic analysis, is sister to the remaining *Neohermes* species. Therefore, the present finding of a new species is key to understanding the evolution of this enigmatic genus. In the phylogeny of world fishfly genera reconstructed by Liu et al. [29], *Neohermes* belongs to a monophyletic group, which also includes *Protochauliodes* (distributed in eastern Australia, Chile, and western North America), *Taeniochauliodes* Esben-Petersen (endemic to South Africa), and *Nothochauliodes* Flint (endemic to Chile). This group is supported by the partial coalescence between the stem of 1A and anterior branch of 2A in the forewing. Based on morphology, *Neohermes* is considered to be the sister group to *Protochauliodes*, whose status however is still questionable [29]. Penny predicted that the ancestral *Protochauliodes* might have originated in Gondwana and dispersed northward to western North America [30]. However, both present and previous ancestral area reconstructions [29] involving this genus suggest that either western North America or a broader area comprising western North America and austral regions (e.g. eastern Australia and South America) might be the range of the ancestor of *Neohermes* + *Protochauliodes*. On one hand, if these two genera diverged in western North America, the aforementioned northward movement of ancestral *Protochauliodes* will be falsified. Thus, the occurrence of some *Protochauliodes* species from austral regions implies that the divergence between *Neohermes* and *Protochauliodes* might have taken place before the breakup of Laurasia and Gondwana during the Early-Middle Jurassic. On the other hand, if *Neohermes* and *Protochauliodes* were separated in a broad area covering western North America and some austral landmasses, this divergence event is likely to be correlated to the splitting between Laurasia and Gondwana. In this case, the western North American species of *Protochauliodes* might have diversified after the northward dispersal of their common ancestor from Gondwana. The most significant event of faunal exchange between South and North America (post separation ca. 175 MYA) was facilitated by the formation of the Central American land bridge approximately 3.5 MYA [31]. However, compared with the Mesozoic origin of these two genera, such Pliocene dispersal of *Protochauliodes* needs further consideration.

*Neohermes* is the only fishfly genus distributed in both eastern and western North America. Based on our ancestral area reconstruction, the initial divergence within *Neohermes* was estimated to be confined to western North America, and a dispersal event was recognized from western North America to western plus eastern North America, which was the distribution area for the common ancestor of the five *Neohermes* species except *N*. *inexpectatus* sp. nov. The current reconstructed geological history of North America from the Mesozoic to the present shows that the Mid-Continental Seaway separated western and eastern North America in the Mid-Late Cretaceous (100–80 MYA) and finally disappeared at the end of the Cretaceous (70 MYA). This was presumably followed by an Early Cenozoic period of floral and faunal exchange between the eastern and western Nearctic [32]. A compatible pattern of the above biogeographic and the geological scenario could be that the initial divergence within *Neohermes*, perhaps also the origin of this genus, took place when western North America was isolated from eastern North America during the Mid-Late Cretaceous. Subsequently, the closure of the Mid-Continental Seaway at the end of the Cretaceous facilitated the eastward dispersal of *Neohermes*. However, as discussed above, if *Neohermes* and *Protochauliodes* diverged around or even before the splitting of Laurasia and Gondwana, one cannot deny the past distribution of ancestral *Neohermes* in eastern North America preceding the divergence of modern species. Likewise, the modern eastern species might be re-introduced by dispersal after assumed extinction of some ancestral species in eastern North America. Hence, dating the origin of *Neohermes* is crucial to clarify the early speciation process of this genus, which however has to be done in future molecular-clock model-based studies.

The separation between the eastern and western modern species of *Neohermes* appears to be due to the uplift of the Cordilleran Mountain System, which was presumably associated with massive vicariance in Nearctic biotas [32]. Nevertheless, there had been two major orogenic phases of the Cordilleran System, respectively occurring in the Late Eocene (35 MYA) and the Late Oligocene (25 MYA). Between these two orogenic events, the early Cordilleran System had been completely wiped out during the Early Oligocene (30 MYA) [32]. Without a molecular divergence time estimation, we can only predict that the eastern and western extant species of *Neohermes* diverged no later than the second formation of the Cordilleran System.

Considering the biogeography of the two western species, i.e. *N*. *californicus* and *N*. *filicornis*, their distributional range is respectively along the Sierra Nevada and the Pacific Coast Ranges, suggesting possible allopatric speciation correlated with the isolation of these two close but independent mountain chains. Inferring the biogeographic scenario associated with the diversification of the three eastern species, however, is more difficult because no clear geographic barrier has yet been found among the distribution areas of these species. The only notable issue is that the separation of *N*. *matheri* from Mississippi and *N*. *angusticollis* from Georgia, South Carolina and North Carolina might be attributed to the faunal discontinuities repeatedly uncovered between Atlantic and Gulf Plains [33], with a drainage system, which includes the Apalachicola River Basin and the Flint and Chattahoochee Rivers, as the often-recovered boundary for the vicariance [34].

## Conclusions

The present new fishfly species from North America is a remarkable finding considering that the taxonomy of Nearctic Megaloptera has been extensively studied previously. Our phylogenetic study demonstrates that *N*. *inexpectatus* sp. nov. is the sister to other *Neohermes* species and the biogeographic analysis suggests a Mesozoic origin of the genus. It would be of great interest to date the origin and interspecific diversification of such a relictual aquatic insect group using a molecular approach so as to better understand the evolutionary history of this genus, as well as the Nearctic Megaloptera. In addition, populations of *N*. *inexpectatus* sp. nov. could be endangered or even close to extinction owing to the scarcity of collecting records, especially recently. It is therefore necessary to do intensive surveys around the known distributional area of *N*. *inexpectatus* sp. nov. in order to determine its current conservation status.

## Materials and Methods

### Ethics statement

All insect specimens for present study were acquired from authorized museum collections but no newly collected in the field. No species in the genus *Neohermes* are included on the ‘‘List of Protected Animals in U.S.A.”.

### Material examined

The type specimens of the presently described new species are deposited in the California State Collection of Arthropods (CSCA), California Department of Food & Agriculture (CDFA), Sacramento, CA, U.S.A. and the California Academy of Sciences (CASC), San Francisco, CA, U.S.A. Other specimens (S1 File) used for drawings and phylogenetic analysis are deposited in the Natural History Museum (BMNH), London, U.K.; the Entomological Museum, China Agricultural University (CAU), Beijing, China; the National Museum of Nature and Science (NSMT), Tsukuba, Japan; the National Museum of Natural History (USNM), Smithsonian Institutions, Washington, D.C., U.S.A.

Genitalia preparations were made by clearing the apex of the abdomen in cold, saturated KOH for 8–10 h. After rinsing the KOH with acetic acid and water, the apex of the abdomen was transferred to glycerin for further dissection and examination. Following examination genitalia were stored in fresh glycerin in a microvial pinned below the specimen. The terminology of the genitalia follows that of Liu et al. [35].

The electronic edition of this article conforms to the requirements of the amended International Code of Zoological Nomenclature, and hence the new names contained herein are available under that Code from the electronic edition of this article. This published work and the nomenclatural acts it contains have been registered in ZooBank, the online registration system for the ICZN. The ZooBank LSIDs (Life Science Identifiers) can be resolved and the associated information viewed through any standard web browser by appending the LSID to the prefix “http://zoobank.org/”. The LSID for this publication is: urn:lsid:zoobank.org:pub: 8E74FFA1-D10B-4D8B-ACE3-AC59F08E7097. The electronic edition of this work was published in a journal with an ISSN, and has been archived and is available from the following digital repositories: PubMed Central, LOCKSS.

### Phylogenetic analysis

Eighteen morphological characters listed below were coded for six ingroup taxa and two outgroup taxa, with 16 binary and two multistate (S2 File).

1. Male antennae: (0) filiform; (1) moniliform.
2. Male antennae with whorl of long setae: (0) absent; (1) present. In Chauliodinae the moniliform male antennae with whorl of long setae are only present in all *Neohermes* species. The state 1 of above two characters attributes to the autapomorphies of this genus.
3. Male antenna, length: (0) shorter than 1/2 length of forewing; (1) about 3/5 length of forewing; (2) about 3/4 length of forewing. The male antenna in *Dysmicohermes* is shorter than 1/2 length of the forewing, while in *Protochauliodes* and *Neohermes* the male antennae are much longer and respectively reach to about 3/5 and 3/4 length of the forewing.
4. Forewing with MA: (0) bifurcated; (1) simple. The basically bifurcated MA of the forewing is considered to be plesiomorphic [29]. State 1 herein is present in *Protochauliodes* and all *Neohermes* species.
5. Forewing with 1A and 2A: (0) attached by a crossvein; (1) attached by a fusion for a short distance. The anterior branch of 2A is fused with the stem of 1A for a short distance in the forewings of four fishfly genera, including *Protochauliodes*, *Neohermes*, *Taeniochauliodes*, and *Nothochauliodes*, and this state is considered to be apomorphic [29].
6. Hindwing with posterior branch of MP: (0) bifurcated; (1) simple. The state 1 is considered to be apomorphic [29] and herein present in *Protochauliodes* and all *Neohermes* species.
7. Male gonocoxites 9: (0) not fused with gonocoxites 10; (1) fused with gonocoxites 10. In most fishfly genera except *Dysmicohermes* and *Orohermes*, the remnant of reduced male gonocoxites 9, which is usually setose, is present but fused with the lateral arms of gonocoxites 10 (Fig 2C).
8. Male ectoproct distally with conical ventral lobe: (0) absent; (1) present. State 1 is present in *N*. *californicus* and *N*. *filicornis* (Fig 4J and 4M).
9. Male ectoproct with spinous setae: (0) present along inner margin; (1) confined to inner portion of apex. State 1 is present in *N*. *californicus*, *N*. *filicornis* and *N*. *inexpectatus* sp. nov. (Fig 2B).
10. Male gonocoxites 10: (0) paired; (1) unpaired. In Chauliodinae state 1 is considered to be apomorphic and present in most genera except *Dysmicohermes* and *Orohermes*.
11. Male gonocoxites 10 with median plate: (0) nearly as long as lateral arm; (1) much longer than lateral arm. In outgroup species, median plate of male gonocoxites 10 is relatively short and nearly as long as lateral arm. However, in most species of *Neohermes*, the median plate of male gonocoxites 10 is prolonged and much longer than the lateral arm (Fig 4). Remarkably, *N*. *inexpectatus* sp. nov. has a rather short median plate of male gonocoxites 10 (Fig 2D).
12. Male gonocoxites 10 with lateral margins of median plate: (0) nearly straight; (1) convexed. In most *Neohermes* species except *N*. *inexpectatus* sp. nov., the median plate of male gonocoxites 10 is convexed on lateral margins (Fig 4), which is considered to be apomorphic.
13. Male gonocoxites 10 with prolonged subtriangular median plate: (0) absent; (1) present. Among the *Neohermes* species, the median plate of male gonocoxites 10 is remarkably elongated and subtriangular in *N*. *angusticollis* and *N*. *matheri* (Fig 4C and 4I).
14. Genital papillae: (0) absent; (1) present. The genital papillae are a pair of rugose and membranous lobes beneath male gonocoxites 10. Presence of this character is considered to be plesiomorphic by Glorioso [36] but interpreted to be apomorphic by Contreras-Ramos [37]. We agree with Contreras-Ramos [37] and code the presence of genital papillae to be apomorphic. In Chauliodinae, the genital papillae are only present in quite a few genera, e.g. *Neohermes* and *Protochauliodes*.
15. Female gonocoxites 8 with posterior margin: (0) convexed; (1) feebly concaved; (2) strongly concaved. The shape of female gonocoxites 8 more or less differs among fishfly genera and species. In *Dysmicohermes* and *N*. *inexpectatus* sp. nov., the posterior margin of this sclerite is distinctly convexed, which is considered to be plesiomorphic. In the remaining species, the posterior margin of female gonocoxites 8 is concaved, and it is conspicuously concaved, forming a broad V-shaped incision in *N*. *angusticollis* and *N*. *matheri* (Fig 5B and 5F).
16. Female gonocoxite 9 distally with a small subtriangular projection: (0) absent; (1) present. State 1 is only present in three *Neohermes* species, i.e. *N*. *angusticollis*, *N*. *concolor*, and *N*. *matheri* (Fig 5).
17. Female gonostyli 9: (0) present; (1) absent. State 1 is herein present in two ingroup species, i.e. *N*. *californicus* and *N*. *filicornis* (Fig 5G and 5I).
18. Broad membranous area between female gonocoxites 8 and abdominal segment 9: (0) absent; (1) present. State 1 is herein present in three *Neohermes* species, i.e. *N*. *angusticollis*, *N*. *concolor*, and *N*. *matheri* (Fig 5A, 5C and 5E).

*Dysmicohermes* Munroe and *Protochauliodes* Van der Weele were selected as outgroups because *Dysmicohermes* belongs to the basal-most fishfly lineage and *Protochauliodes* is considered to be the sister group of *Neohermes* [29]. The analysis was performed using NONA ver. 2.0 [38] with a heuristic search. The heuristic search was used with maximum trees to keep setting to 10000 and number of replication setting was 100. We also analyzed the datasets using TNT ver. 1.1 [39] with an initial New Technology search set to 100 (using a driven search with sectorial search, ratchet, drift, and tree fusing; finding the minimum tree 10 times). The branch support values were calculated with the function implemented in TNT (TBR from existing trees, retain trees suboptimal by 10 steps). All characters were treated as unordered and with equal weight. Character states were mapped on a most parsimonious tree (MPT) using WinClada ver. 1.00.08 [40], showing only unambiguous changes.

### Ancestral area reconstruction

As the ingroup species are disjunctively distributed in eastern and western North America, we defined these two areas (i.e. western North America (a) and eastern North America (b)) as the areas of endemicity used for the ancestral area reconstruction. In addition, southern South America (c) and eastern Australia (d) were also defined as the endemic areas because the outgroup *Protochauliodes* also occurs in these two areas besides western North America. Ancestral areas at internal nodes were inferred using a dispersal-vicariance (DIVA) optimization model [41] implemented in the program RASP2.0 Beta [42]. The DIVA optimization was conducted with default settings with the maximum number of areas in ancestral ranges constrained to three.

## Supporting Information

S1 File Materials examined for previously described species of *Neohermes*. (DOC)

S2 File Character matrix. (TXT)
