# Supplementary material for: A New Fishfly Species (Megaloptera: Corydalidae: Neohermes Banks) Discovered from North America by a Systematic Revision, with Phylogenetic and Biogeographic Implications
Source: PLoS One. 2016 Feb 17;11(2):e0148319. doi: 10.1371/journal.pone.0148319 (PMC4757407; doi:10.1371/journal.pone.0148319)
Supplement: S1 File — (DOC) [file pone.0148319.s001.doc]

**File S1.** Materials examined for previously described species of *Neohermes*.

***Neohermes angusticollis* (Hagen, 1861)**

Neotype ♂, U.S.A., Georgia: Atlanta, 11.VI.1939, P.W. Fattig (USNM). 1♀, U.S.A., Georgia, White Co., Unicoi State Park, 14.VI.1987, S.W. Gross (USNM); 2♂, U.S.A., North Carolina (BMNH).

***Neohermes californicus* (Walker, 1853)**

2♂1♀, U.S.A., California, Antler, Shasta, 22.VII.1990, J.B. & G.M. Ward (BMNH).

***Neohermes concolor* (Davis, 1903)**

1♂, U.S.A., Virginia, Great Falls, 25.VI.14, R.P. Currie (NSMT); 1♀, U.S.A., Washington D.C., 25.VI.14, R.P. Currie (NSMT); 1♂1♀, U.S.A., Arkansas, Wash Co., Devil’s Den St. Pk., 19.VI./5.VII.1966, R.W. Hodges (CAU).

***Neohermes filicornis* (Banks, 1903)**

1♂, U.S.A., Arizona, Coconino Co., West Fork, 6500’, 16 mi SW Flagstaff, 8.VII.1961, J.G. Franclemont (USNM); 1♂, U.S.A., New Mexico, nr. Silver City, 9.VII.1963, P.J. Spangler (CAU); 1♀, U.S.A., California, San Diego Co., NAS Miramar 9, 19.V.1997, N. Bloomfield (CAU).

***Neohermes matheri* (Flint, 1965)**

Holotype ♂, U.S.A., Mississippi, Hinds Co., Clinton, 12.VI.1960 (USNM). 1♂1♀, U.S.A., Mississippi, Warren Co., Bovina, 30.V.1973 and 13.VI.1972 (USNM).
